# Supplementary material for: Salvage External Beam Radiotherapy after Incomplete Transarterial Chemoembolization for Hepatocellular Carcinoma: A Meta-Analysis and Systematic Review
Source: Medicina (Kaunas). 2021 Sep 22;57(10):1000. doi: 10.3390/medicina57101000 (PMC8539441; doi:10.3390/medicina57101000)
Supplement: Supplementary file 1 [file medicina-57-01000-s001.zip › medicina-1380720-supplementary.pdf]

Supplement Table S1. Scoring sheet according to Newcastle-Ottawa Scale

	Selection				Comparability	Outcome			**Overall score (9 to be full)**
	1	2	3	4	1	1	2	3	
	Representativeness of the exposed cohort	Selection of the non exposed cohort	Ascertainment of exposure	Outcome of interest was not present at start of study	Comparability of cohorts on the basis of the design or analysis	Assessment of outcome	Was follow-up long enough for outcomes to occur	Adequacy of follow up of cohorts	
Oh	1	1	1	1	0	1	1	1	7
Kim	1	1	1	1	0	1	-1	1	6
Choi	1	1	1	1	0	1	1	1	7
Kang	1	1	1	1	0	1	1	1	7
Shim	1	1	1	1	0	1	1	1	7
Zhong	1	1	1	1	0	1	1	1	7
Chiang	1	1	1	1	0	1	1	1	7
Jacob	1	1	1	1	0	1	1	1	7
Kibe	1	1	1	1	0	1	1	1	7
Yao	1	1	1	1	0	1	1	1	7
Byun	1	1	1	1	0	1	1	1	7
Park	1	1	1	1	0	1	-1	1	6
